# Innovative use of magnetic resonance imaging-guided focused ultrasound surgery for non-invasive breast cancer: a report of two cases

**DOI:** 10.1186/s40792-020-01032-3

**Published:** 2020-11-23

**Authors:** Akiko Matsutani, Yoshimi Ide, Sakiko Miura, Masafumi Takimoto, Sadao Amano, Seigo Nakamura

**Affiliations:** 1 Division of Breast Surgical Oncology, Department of Surgery, Showa University School of Medicine, 1-5-8 Hatanodai Shinagawa-ku, Tokyo, Japan; 2 Division of Breast Surgical Oncology, Shinntoshinn Musashino Clinic, 2-389-1 Kitabukurocho Omiya-ku, Saitama, Japan; 3 Department of Pathology, Showa University School of Medicine, 1-5-8 Hatanodai Shinagawa-ku, Tokyo, Japan

**Keywords:** MRgFUS, Breast cancer, Non-invasive technique, Ablation, High-intensity focused ultrasound

## Abstract

**Objective:**

This report describes the first clinical experience with magnetic resonance imaging-guided focused ultrasound surgery (MRgFUS) using the ExAblate 2100 system for non-invasive breast cancer.

**Methods:**

Two women with non-invasive breast cancer underwent MRgFUS treatment. One week after the MRgFUS treatment, US-guided vacuum-assisted biopsy was performed for the ablated lesions at the same time as breast-conserving surgery.

**Results:**

The patients experienced good cosmetic outcomes and did not experience any severe adverse events, such as skin burns. Pathological examination of the surgical specimens revealed a few degenerated intraductal lesions around the breast biopsy markers.

**Conclusion:**

Performing MRgFUS with the new ExAblate 2100 system appears to be safe and feasible. The histopathological results revealed that adequate ultrasound energy in the appropriate location can induce tumor necrosis.

## Introduction

Breast cancer treatment has focused on treatment escalation to improve clinical outcomes. However, ductal carcinoma in situ (DCIS) has generally mild biological characteristics and treatment de-escalation, such as active surveillance, has recently become more prevalent for managing DCIS [1–6]. In this scenario, minimally invasive techniques have attracted attention as alternatives to lumpectomy, such as radiofrequency ablation, cryosurgery, and high-intensity focused ultrasound (HIFU) [7]. Among these techniques, magnetic resonance-guided focused ultrasound surgery (MRgFUS) is a HIFU method that has been used to facilitate targeted drug delivery and to treat uterine fibroids, essential tremor, and desmoid tumors [8–10]. In 2016, Peek et al. evaluated US-guided HIFU for benign tumors based on the change in tumor volume [11]. US-guided HIFU is suitable for benign and focal tumors, but has limited effectiveness for breast tumors that are difficult to evaluate using US.

MRI-guided HIFU is a truly noninvasive procedure that provides closed-loop therapy with accurate tumor location and real-time thermal monitoring. The combination of magnetic resonance imaging with focused ultrasound surgery (MRgFUS) allows the operator to precisely and anatomically determine the lesion’s size and volume, select the appropriate ablation area, and perform real-time temperature monitoring [8]. Additional advantages of MRgFUS include eliminating the need for general anesthesia and hospitalization, avoiding breast deformation, and good cosmetic outcomes. Various types of MRgFUS devices have been introduced [1–6]. In 2001, Huber et al. reported the first case of MRgFUS treatment for a 56-year-old patient with a 2.2-cm invasive ductal carcinoma, which was performed using a 1.5-T MRI and US transducer system that achieved pathologically complete ablation of the tumor [12]. Merckel et al. evaluated MRgFUS using the Sonalleve breast platform with 1.5 T MRI, which revealed that temperature during sonication was not related to the area of pathological necrosis, and that the sonication power varied according to the proportion of fat and mammary gland [5]. However, this result requires validation among Japanese women, who have relatively small amounts of fat [13]. Relative to in previous reports, our center uses two new instruments: 3-T MRI and the ExAblate 2100 system. The 3-T MRI system provides high-resolution imaging and enables us to more accurately evaluate the spreading of breast cancer, relative to 1.5 T MRI [14]. The ExAblate 2000/2100 system (InSightec, Haifa, Israel and Dallas, TX) is the most widely recognized MRgFUS device and has been approved by the US Food and Drug Administration for treating essential tremor, uterine fibroids, bone metastases, and adenomyosis. This system is joined to an MRI scanner (GE Medical systems, Milwaukee, WI). The present study evaluated the ExAblate 2100 transducer system, which has several improvements relative to the ExAblate 2000 system. For example, the ExAblate 2100 system can move in the vertical and horizontal directions, which can reduce the energy density on non-target regions, such as the patient’s skin and nerves. Second, we can control the transducer aperture, which allows for freer ablation of the target area. Third, this system uses a computer-assisted ablation system that automatically continues sonication, which may help shorten the treatment time. Finally and most importantly, minor body movements does not lead to automatic stopping of the ablation. Therefore, the ExAblate 2100 system may be effective while reducing damage to non-targeted tissues. A previous report has indicated that MRgFUS treatment using the ExAblate 2000 system is safe and effective for invasive breast cancer [1–4, 15]. However, few studies have evaluated the safety and feasibility of MRgFUS using the ExAblate 2100 system for non-invasive breast cancer, which may be managed using conservative or delayed treatment. We report the first clinical experience using MRgFUS with the ExAblate 2100 system and a 3-T MRI scanner as treatment for non-invasive breast cancer.

## Patients and methods

### Study overview

This study evaluated two patients with non-invasive breast cancer who provided informed consent at the Showa University. The patients received an explanation regarding two potential benefits. First, their participation might help guide medical innovation that could be implemented in future clinical practice. Second, most DCIS cases involve no palpable lesion, although the ablated lesion becomes palpable after MRgFUS treatment. Thus, undergoing this treatment can help guide decision-making regarding the resection area, which can improve the likelihood of negative margins after breast surgery.

All patients had undergone enhanced MRI to confirm the target lesion’s location. The MRgFUS treatment was performed in the Shintoshinn-musashino clinic with the patient in the prone position under local anesthesia. Treatment progress was monitored from a MR workstation, and enhanced MRI was performed immediate after the ablation to evaluate tissue changes. At the end of the MRgFUS treatment, we evaluated patient’s breast deformation using the scoring method proposed by the Japanese Breast Cancer Society Sawai group [16]. This method was based on eight items: breast size, breast shape, scar, breast firmness, size/shape of nipple and areola, color tone of nipple and areola, nipple position, and position of the inframammary fold.

One week after the MRgFUS, the patients underwent US-guided vacuum-assisted biopsy (post-MRgFUS biopsy) before surgical resection at Showa University. The post-MRgFUS biopsy helped determine whether breast surgery could be omitted, based on the presence or absence of viable tumor in the post-ablation biopsy specimens. After surgery, patients received adjuvant radiation therapy according to the clinical guidelines. The post-MRgFUS biopsy and surgical specimens were pathologically assessed using hematoxylin and eosin staining (approximate thickness: 5 mm). Tumor necrosis was identified based on the presence of thermal erythrocyte coagulation. We also monitored for any adverse events during the study period. The primary endpoint of this study was to evaluate complete tumor necrosis of the ablated area by histopathological methods. The secondary endpoint was to assess the safety of MRgFUS treatment and to evaluate the degree of deformation.

### Inclusion and exclusion criteria

The inclusion criteria were:

- Women who were > 18 years old, were diagnosed with non-invasive breast cancer, and provided informed consent.
- The targeted lesion was a focal mass with the diameter of ≤ 2 cm and a distance of > 1 cm from the ribs, nipple, and skin.
- Ability to undergo MRI.

The exclusion criteria were:

- Women with metallic implants or other factors that precluded MRI.
- Pregnancy or lactation.
- Women who were not eligible for this study because of other reasons.

### Patients

Two patients were recruited for this study. All procedures were approved by the institutional review board of Showa University (November 21, 2017; approval number: 2352) and with the 1964 Helsinki Declaration and its later amendments. Both patients provided written informed consent before participating. The study protocol was registered with the UMIN Clinical Trials Registry (UMIN000030255; December 1, 2017).

The first patient was a 70-year-old woman with mammographic abnormalities that were detected during a medical examination. No physical findings or family history of breast or ovarian cancers were reported. Mammography revealed a group of amorphous calcifications in the right breast and ultrasonography revealed an approximately 25-mm irregularly shaped hypoechoic non-mass lesion in the left breast. Enhanced MRI revealed a 6-mm oval-circumscribed mass with an early peak, a delayed washout pattern, and heterogeneous enhancement in the right breast. Mammogram-guided vacuum-assisted breast biopsies of the targeted lesion revealed highly suspicious findings, which supported a diagnosis of apocrine type DCIS.

The second patient was a 69-year-old woman with an MRI-detected left breast tumor. She had a history of right breast cancer that had been treated using mastectomy and axial lymph node dissection. No family history of breast or ovarian cancer was reported. Mammography revealed an irregularly shaped indistinct high-density mass in the left breast, although ultrasonography revealed no specific findings. Enhanced MRI revealed a 12-mm lobulated-circumscribed mass in the left breast, and MRI-guided vacuum-assisted breast biopsies revealed an intraductal proliferative lesion with diffuse positivity for estrogen receptor and progesterone receptor. These results supported a suspicion of malignancy and a high possibility of the lesion progressing to cancer, given the patient’s history of breast cancer.

### MRgFUS treatment

**Fig. 1:**
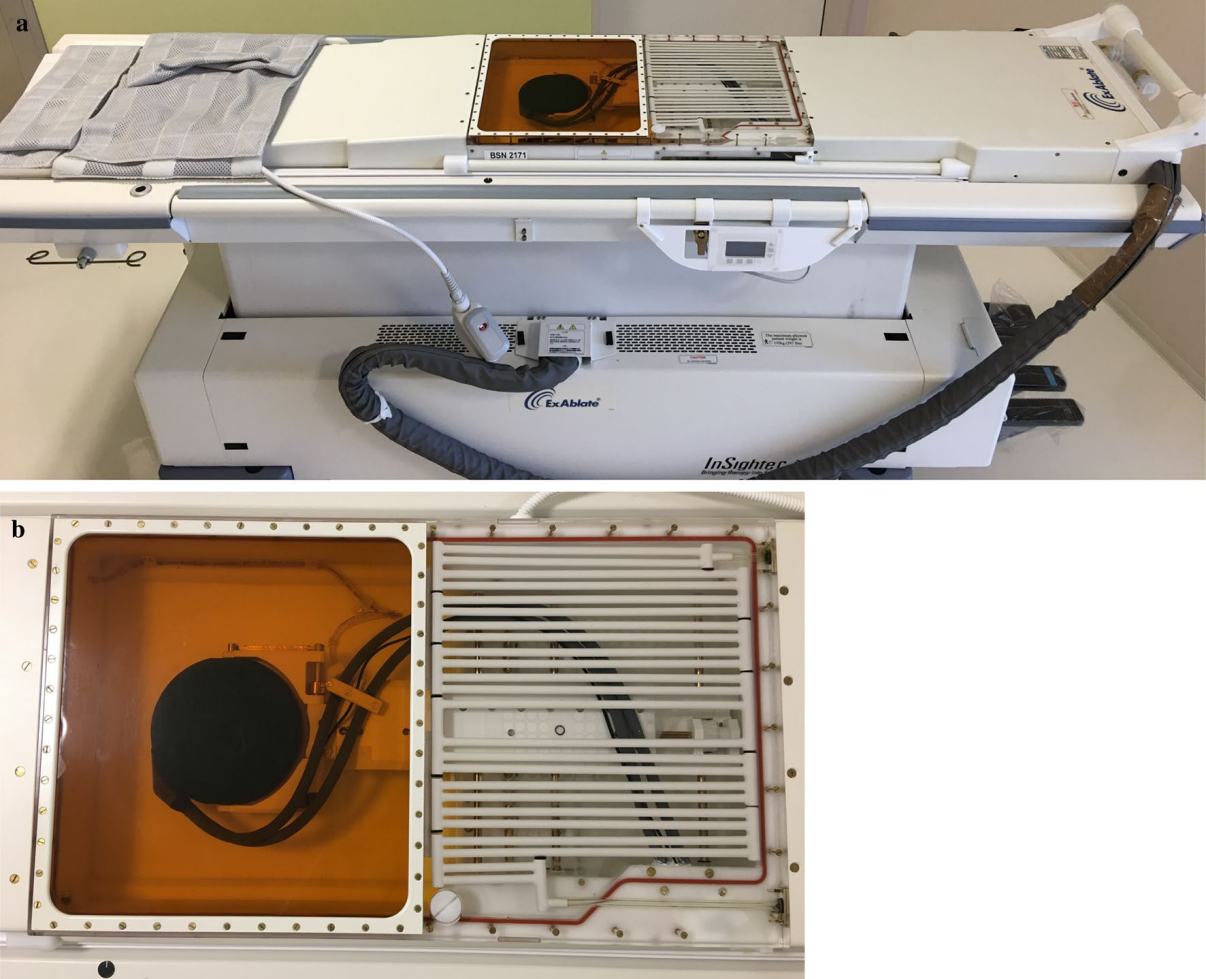
The ExAblate 2100 treatment table. **a** Photograph shows the patient treatment table. Before treatment, this table is connected to the MRI scanner. **b** Photograph shows FUS transducer and the positioning device of the FUS transducer. During treatment, the FUS transducer moves to the appropriate area for sonicating the targeted lesion

**Fig. 2:**
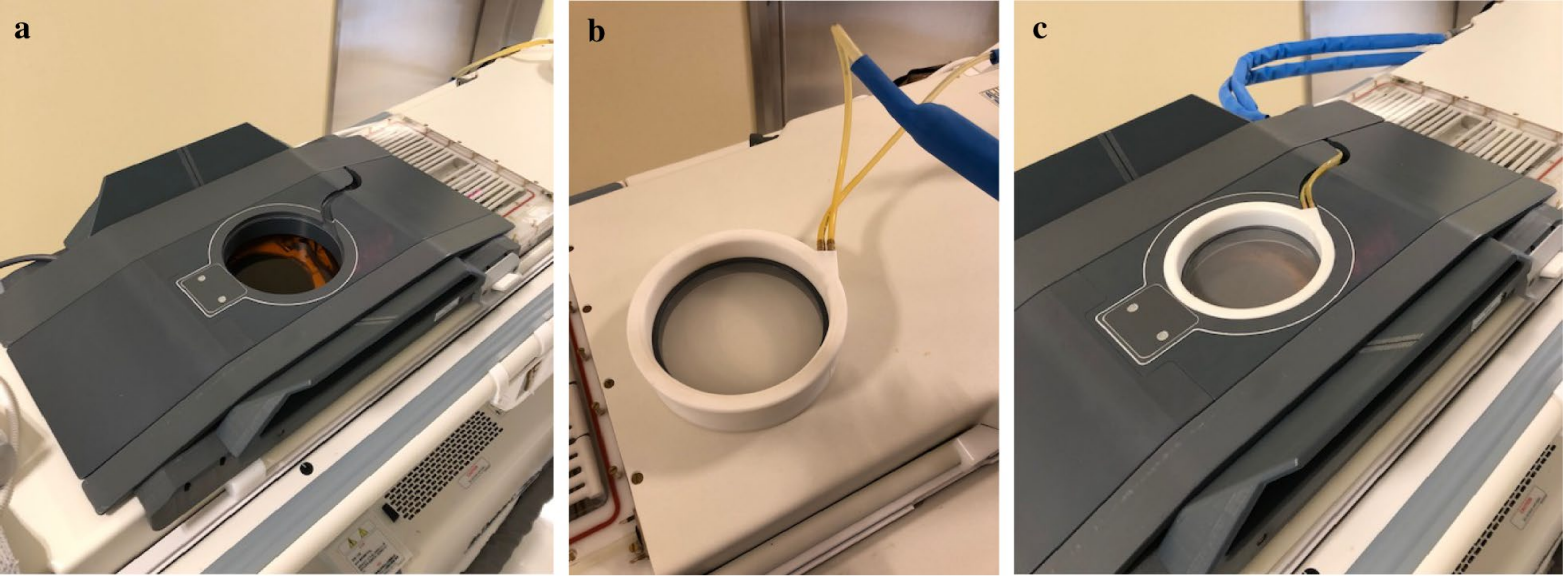
Details of the ExAblate 2100 Breast coil and set up of the FUS table. **a** Breast coil table is placed on the patient’s treatment table. **b** Cooling coil insert in the center hole of the breast coil table. Cooling coil inner diameter is 152 mm, coil outer dimensions are 650 × 195 mm, coil height is 39 mm, and weight of cooling coil is 6.4 Ibs. **c** Patients were placed in the prone position on the FUS treatment table with the targeted breast placed in the water-filled cooler

**Fig. 3:**
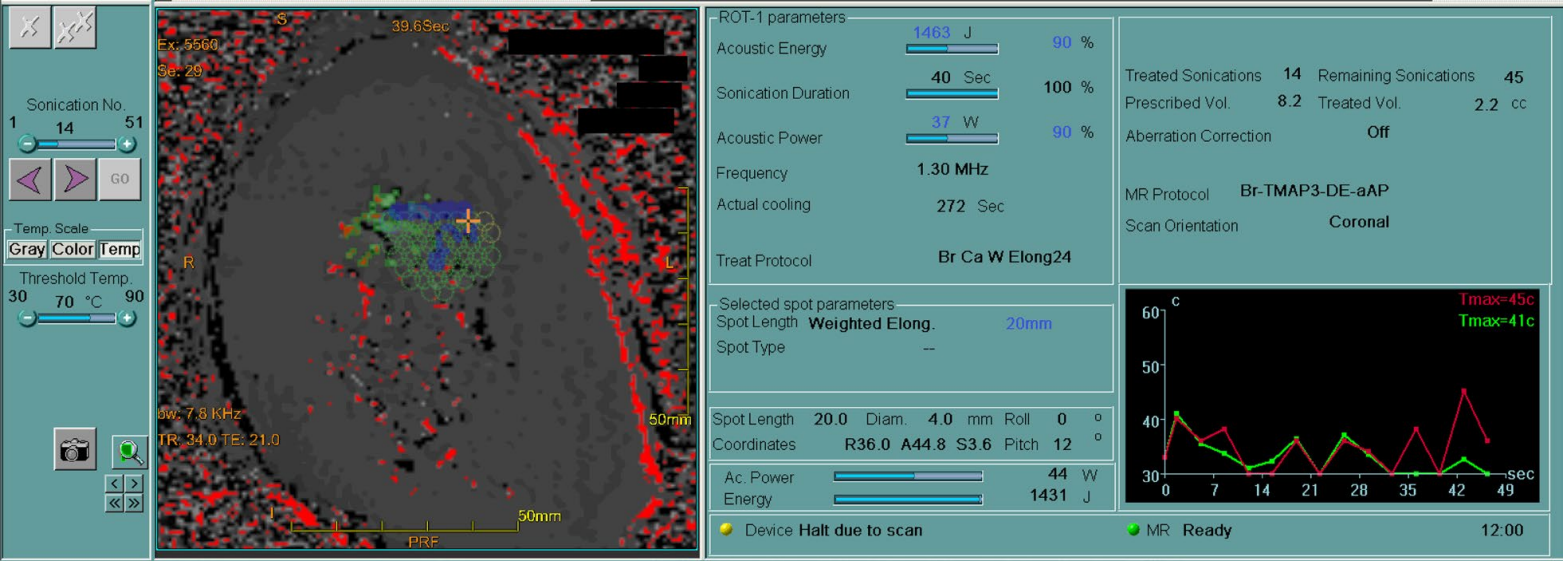
This figure shows the thermal map during treatment of case 1. Left: sonication area is depicted by small honeycomb structural spots. Green and blue spots indicate before and after sonication, respectively. Right: line graph shows sonication temperature on the cursor. Red and green lines indicate theoretical and actual temperature on the cursor, respectively

The ExAblate 2100 system consists of an operating console, treatment table, breast coil, device cabinet, and cooling device. The system was stored and operated using the conditions shown in Table 1. The operators monitored the treatment process via an MR workstation and the operating console. The treatment table contains an FUS transducer and a positioning device to control the FUS transducer (Fig. 1). The breast coil was placed on the treatment table and the patient lay in the prone position with the affected breast in the water-filled cooling coil, which contains cold water that is circulated by the cooling device (Fig. 2). The cooling coil ensures the water does not directly contact the patient’s skin, but still provides sufficient cooling to avoid skin redness or burns. Intravenous local sedation was used during the treatment to minimize unexpected body and breathing movements. The sonication area is depicted by small honeycomb structures, with green spots indicating structures before sonication and blue spots indicating structures after ablation. A line graph of the thermal map was used to automatically display the sonication temperature at the cursor (Fig. 3).

**Table 1:** Environmental requirements of ExAblate 2100 system

	Storage	Operating conditions
Humidity	20% to 95%	45% to 75%
Barometric pressure	86 kPa to 106 kPa	
Temperature	− 15 °C to 55 °C	15 °C to 35 °C

## Results

### Case 1

**Fig. 4:**
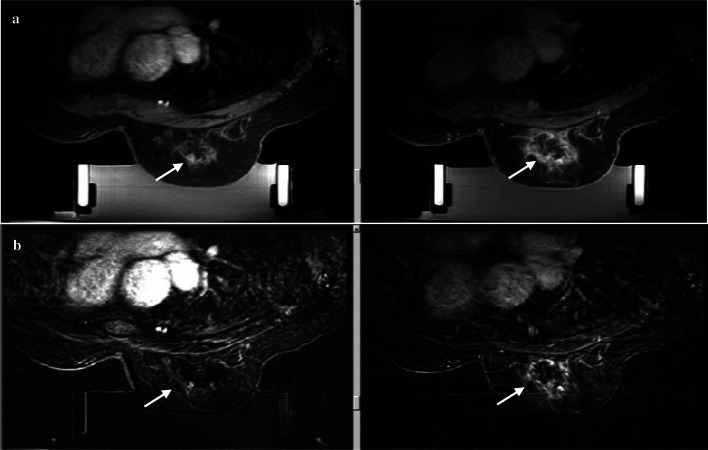
Enhanced magnetic resonance imaging (MRI) before and after MRgFUS treatment. Low-intensity area after ablation indicated tissue necrosis. High-intensity area indicated edema change of the tissue. White arrows indicate the change of the ablated area before and after MRgFUS treatment. **a** T1-weighted imaging of a 70-year-old-woman before (left) and after (right) ablation. **b** T1-weighted imaging of a 69-year-old-woman before (left) and after (right) ablation

**Fig. 5:**
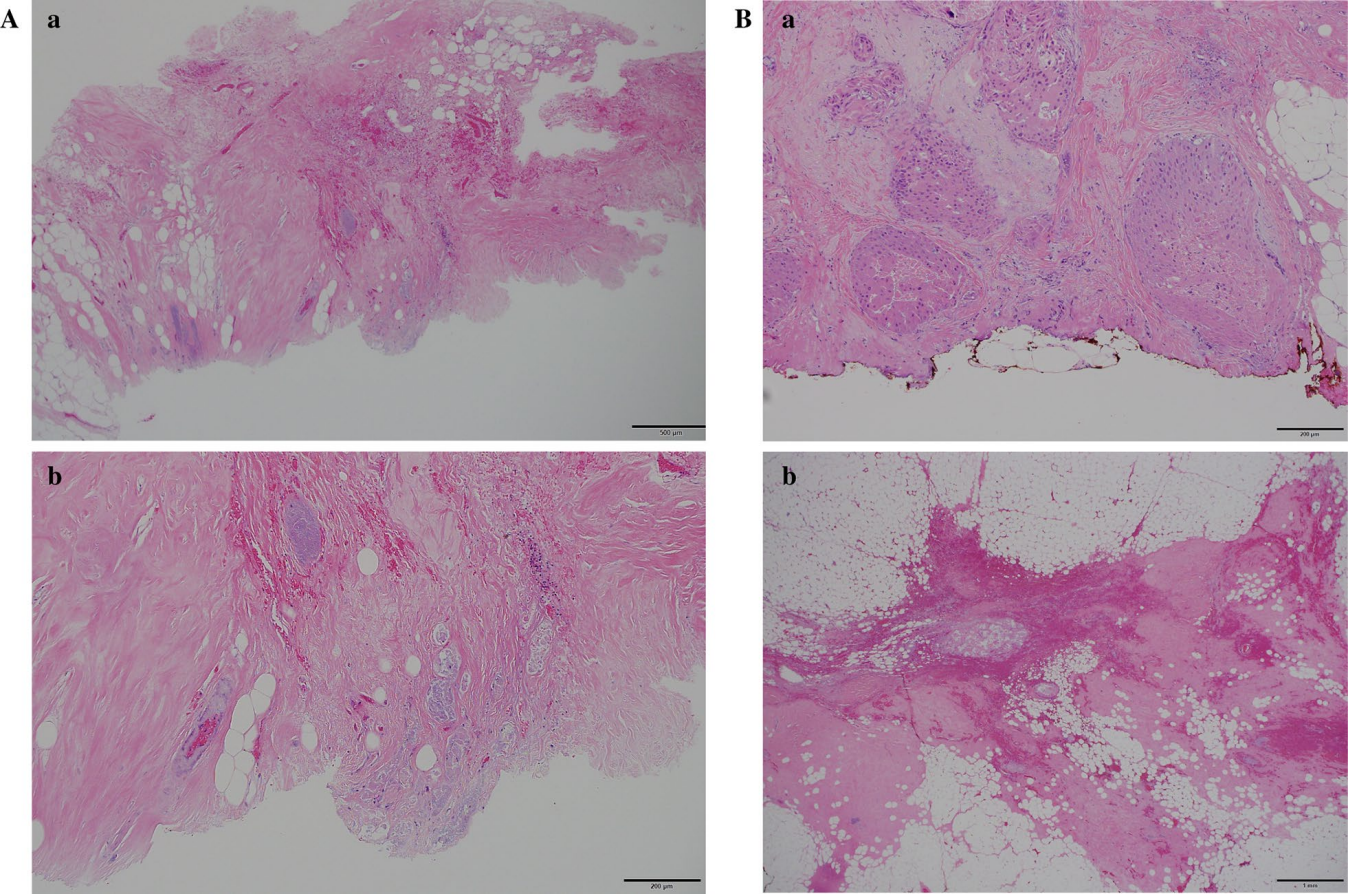
Histopathology of a 70-year-old-woman. **a** H&E staining of the biopsy specimen of the ablated area. (a) A few duct-like structures and stroma with hemorrhage. (b) At higher magnification, several ducts are severely damaged and filled with only necrotic debris. **b** H&E staining of the excised specimen of ablated area. (a) Necrotic-like changes are seen in every duct. No viable tumor cells in the specimen. (b) The area around the tissue marker clip. A few thermally damaged ducts and marked stromal hemorrhage

The first patient was a 70-year-old woman with DCIS. During the MRgFUS treatment, each sonication was performed for 20 s followed by 1 min of cooling time to avoid skin burning. Sixty sonication applications were performed, which required a treatment time of 180 min. The average energy during the treatment was 842 J. Enhanced MRI after the treatment revealed a ring-like enhanced lesion that was caused by necrosis and edema related to the ablation (Fig. 4). Histopathology revealed 100% necrosis of the targeted tumor in the post-MRgFUS biopsy specimens and 70% necrosis of the ablated area in the surgical specimens (Fig. 5). This was attributed to increased temperature because of ultrasound reflection around the biopsy clip, which caused breast pain. Thus, inadequate sonication energy was presumably used to avoid patient complaint. No severe adverse events were observed after the MRgFUS treatment, such as skin redness or burns. The patient reported shoulder and breast pain that were related to remaining in the same posture throughout the treatment, as well as the excessive temperature that was caused by irregular ultrasound reflection near the biopsy clip (Table 2). No breast deformation was observed and 12 scores were obtained by Sawai’s scoring method. The patient underwent adjuvant radiation therapy after surgical resection.

**Table 2:** Safety of MRgFUS treatment

Case	Adverse event symptoms	Severity	Cause	Action	Outcome
70-year-old woman	Pain (breast)	Mild	Treatment	Local anesthesia	Resolved
	Shoulder pain	Mild	Posture of patient during treatment	Pain relief medication	Resolved
69-year-old woman	Pain (breast)	Mild	Treatment	Local anesthesia	Resolved

### Case 2

**Fig. 6:**
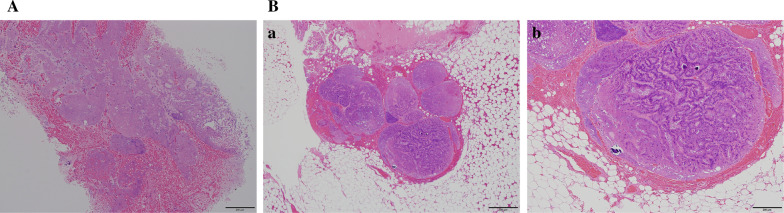
Histopathology of a 69-year-old-woman. **A** H&E staining of the biopsy specimen of the ablated area. Duct-like structures filled with necrotic debris and stroma with marked hemorrhage. **B** H&E staining of the excised specimen of ablated area. **a** Necrotic-like changes are evident in most areas of duct epithelium. **b** In the central area of the duct, there is an apparent transition between viable and subviable tumor cells

The second patient was a 69-year-old woman. During the MRgFUS treatment, 37 sonication applications were performed with an average energy of 1,625 J and a treatment time of 240 min. Enhanced MRI after the MRgFUS treatment revealed that the center of the ablated area had changed to a low-intensity lesion, which reflected tissue necrosis caused by the ablation (Fig. 4). The histopathology findings revealed 100% necrosis of the ablated tumor in the post-MRgFUS biopsy specimens and 40% necrosis of the ablated area in the surgical specimens (Fig. 6). The lower necrosis value in the surgical specimen was also presumably related to inadequate ablation energy that was related to increased temperature caused by ultrasound reflection around the biopsy clip. The patient did not experience skin redness or burns (Table 2), had a good cosmetic outcome (12 scores by Sawai’s scoring method), and completed adjuvant radiation therapy after surgery.

## Discussion

This report describes our first clinical experience with MRgFUS using the ExAblate 2100 system and 3-T MRI to treat non-invasive breast cancer. The results indicate that MRgFUS ablation with the ExAblate 2100 system appears to be safe and feasible. Both patients experienced successful ablation of the target tissue with negative margins, which we believe was related to adequate definition of the DCIS extent based on the 3-T MRI findings. Furthermore, the patients did not experience skin redness or burns, and good cosmetic outcomes were achieved for both patients. This may be related to the theoretical temperatures being higher than the actual temperatures (Table 3), as the protocol is modified based on body and respiratory motions. One patient required treatment interruption because of breast pain.

**Table 3:** Results of sonication temperature during MRgFUS treatment

Case	Sonication temperature	*T*_max_ [°C]	*T*_min_ [°C]	*T*_ave_ [°C]
70-year-old female	Theoretical temperature	289	61	154
	Actual temperature	76	33	49
69-year-old female	Theoretical temperature	138	41	87
	Actual temperature	67	36	45

*T*_max_ = maximum temperature, *T*_min_ = minimum temperature, *T*_ave_ = average temperature

The histopathology findings revealed no viable cancer cells in the ablated area for both patients, although degenerated intraductal lesions remained around the biopsy clip. Thus, we could not confirm complete thermal necrosis of the targeted lesion. This effect may be related to the biopsy clip causing ultrasound reflection that led to increased temperature and breast pain. Besides, low ultrasound energy and short sonication duration may explain the inability to achieve complete ablation. We always use clips when performing mammography-guided biopsy for suspected DCIS, because most DCIS is detected as calcification during mammography and may not be detectable using other modalities, including US [17]. Therefore, clips are indispensable for determining the resection area when the lesion cannot be confirmed by US. The biopsy clips are made from titanium (SenoMark™ Ultra Breast Tissue Marker Ribbon Shape for use with ENCOR® 10G), and are MRI compatible and were eligible for MRgFUS. Surgical clips were not permitted during ablation in Merckel et al.’s study, although we used biopsy clips because they are smaller and less likely to influence MRI findings, relative to surgical clips.

The first patient complained of mild but tolerable breast pain, which made it unclear whether the pain was related to the clip, and we performed the same treatment for the second patient. Given that complete ablation was not achieved around the clip, further research is needed to improve the clinical use of MRgFUS. For example, patients could be selected regardless of tumor size and undergo MRgFUS treatment without insertion of the biopsy clip. Moreover, it would be useful to develop a biopsy clip that does not reflect and absorb ultrasound energy.

Although DCIS is very early stage breast cancer, mastectomy is still performed in most DCIS cases. This procedure creates a mental and physical burden on the patient. Thus, MRgFUS may be a useful alternative to mastectomy if complete ablation can be achieved. Furthermore, we view our results as encouraging, as there was no breast deformation and no need for hospitalization. DCIS can involve several types of malignancy, which should be considered when selecting cases, as low-grade DCIS may not require surgery [18–20]. Hence, we expect that de-escalation of treatment for low-grade DCIS will become more common, and additional data regarding the feasibility of MRgFUS as non-surgical local treatment will be of interest to breast surgeons.

The present study only considered patients with tumor diameters of ≤ 2 cm, which would have limited aggressiveness and effect on the prognosis [20, 21]. However, given that these tumors are difficult to detect, we were only able to recruit two patients. In addition, the MRgFUS facility was far from the patients’ residences, which further complicated the recruitment.

Although several clinical studies of MRgFUS for breast cancer have been reported since 2001, this treatment has not become popular worldwide. There are several barriers to the use of MRgFUS. First, pathological evaluation of the residual tumor after the FUS treatment seems to be difficult, as coagulation and necrotic changes occur in the ablated area. Second, FUS requires a prolonged treatment time that took ≥ 3 h in our study. This is because treatment interruption due to patient complaints, which requires the operators to repeat the treatment planning. Furthermore, each sonication step requires 1 min of cooling time to avoid skin burns. Further studies are needed to address these issues, though MRgFUS using the ExAblate 2100 system and 3-T MRI will provide an advanced non-surgical option for patients with non-invasive breast cancer. Assuming the outcomes are similar to those of surgery, MRgFUS might be the first choice as an alternative to surgical resection. The reasons are as follows: (1) many breast cancer patients hope to avoid breast deformation; (2) MRgFUS does not require hospitalization and may help reduce medical expenses; and (3) the pain after MRgFUS is much milder than after surgery. Thus, MRgFUS may open a new avenue for noninvasive treatment of breast cancer that reduces the mental and physical burdens of treatment.

## Conclusion

This is the first report regarding MRgFUS with a new system for treating non-invasive breast cancer. The results indicate that MRgFUS ablation appears to be safe and to achieve tumor necrosis if adequate ultrasound energy can be used. This technique may open a new avenue for non-invasive or minimally invasive surgeries to treat low-risk breast cancer.

## Data Availability

Not applicable.

## Abbreviations

MRgFUS: Magnetic resonance-guided focused ultrasound surgery
US: Ultrasound
DCIS: Ductal carcinoma in situ
HIFU: High-intensity focused ultrasound
MRI: Magnetic resonance imaging
